# Publication lag in interventional, RCT-based meta-analyses within top-tier general medical journals and the CDSR: a protocol for a meta-epidemiological study

**DOI:** 10.3389/fmed.2026.1803578

**Published:** 2026-04-24

**Authors:** Jia Song, Qin Wang, Chongyang Zhao, Li Li, Deying Kang

**Affiliations:** 1 Department of Evidence-Based Medicine and Clinical Epidemiology, Frontiers Science Center for Disease-related Molecular Network, West China Hospital, Sichuan University, Chengdu, China; 2 Clinical Epidemiology and Evidence-Based Medicine Center, West China Hospital, Sichuan University, Chengdu, China; 3 Chengdu Fifth People's Hospital, Chengdu, China; 4 Center of Biostatistics, Design, Measurement and Evaluation (CBDME), Department of Clinical Research Management, West China Hospital, Sichuan University, Chengdu, China

**Keywords:** meta-analyses, meta-epidemiology, publication lag, systematic reviews, top-tier medical journals

## Abstract

**Introduction:**

Systematic reviews and meta-analyses serve as the cornerstone of clinical guidelines, yet their validity hinges on the currency of the included evidence. The publication lag measured as the interval from the last search date to online publication remains unclear in top-tier general medical journals and the Cochrane Database of Systematic Reviews (CDSR). Existing data are largely outdated and lack exploration of associated factors. Our study aims to fill this gap by quantifying the current publication lag in top-tier general medical journals and the CDSR and identifying its independent predictors.

**Methods:**

This meta-epidemiological study will analyze interventional, RCT-based meta-analyses published in top-tier general medical journals and the CDSR between 2023 and 2025. We will calculate the publication lag, assess compliance with AMSTAR 2 timeliness standards, and compare the performance between top-tier general medical journals and the CDSR. Multivariable regression analysis will be employed to determine independent factors which associated with the extent of publication delay.

**Discussion:**

Our study will systematically quantify the current status and determinants of publication lag in top-tier general medical journals and the CDSR. While our reliance on publicly available dates precludes a granular distinction between author-related revisions and editorial processing durations, this limitation may introduce information bias. Specifically, if certain journals attract more complex reviews requiring extensive author revisions, the observed lag may overstate editorial inefficiency. Conversely, high-performance editorial workflows might mask prolonged author delays. By acknowledging these potential directions of bias, our findings will provide a more nuanced, actionable framework for assessing evidence currency.

**Systematic review registration:**

https://osf.io/cjtk.

## Introduction

Systematic reviews and meta-analyses are generally considered to be the highest level of evidence, which serve as the cornerstone for developing clinical practice guidelines, shaping health policy, and guiding individualized clinical decision-making (1, 2). To ensure scientific validity and clinical relevance, meta-analyses are required to synthesize all currently available, up-to-date evidence (3). The rapid increase in published studies presents a significant challenge to the timeliness of systematic reviews (4).

Publication lag is defined as the time interval between the last literature search and the date of online publication (5). If this delay is prolonged, important newly published trials may not be incorporated into the evidence synthesis (6). This makes the evidence outdated and reduces the reliability of the results, potentially leading to incorrect clinical recommendations (6–8). According to Methodological Expectations of Cochrane Intervention Reviews(MECIR) Standard, searches must be conducted within 12 months prior to publication, with a highly desirable target of within 6 months (9). Similarly, the A Measurement Tool to Assess systematic Reviews (Version 2)(AMSTAR 2) tool identifies the timeliness of the search as a critical indicator of a comprehensive strategy, explicitly requiring that the search be conducted within 24 months of the review’s completion to ensure the adequacy and representativeness of the evidence (10).

Despite the rigorous updating quality standards established by Cochrane (MECIR), it remains unclear whether interventional meta-analyses published in the top-tier general medical journals (NEJM, The Lancet, JAMA, and BMJ) strictly adhere to these timeliness requirements. Meanwhile, given the rapid increase in published studies (8), it is unclear whether Cochrane reviews still strictly comply with their own 12-month search requirement. This needs to be examined using up-to-date data (6).

Previous meta-epidemiological research has consistently demonstrated a significant interval between the last search and publication, which directly affects the timeliness of the evidence. Evidence from large-scale surveys indicates a median publication lag of approximately 8 months, with nearly 20% of reviews featuring searches conducted more than a year prior to their release. Existing studies have mainly focused on describing the delay itself, rather than investigating the factors causing it (6, 11, 12). While Borah et al. identified predictors for the registration-to-publication lag and noted that funded reviews took longer (13), the determinants driving the specific delay from search to publication remain largely unexplored. Little is known about how factors like funding, authorship, and Preferred Reporting Items for Systematic Reviews and Meta-Analyses (PRISMA) compliance affect the timeliness of the top-tier general medical journals and CDSR. Quantifying these predictors is crucial for identifying delays and improving the standard of evidence synthesis.

This study aims to quantify and compare the current status of publication lag among interventional meta-analyses published in the top-tier general medical journals and the CDSR from 2023 to 2025, employing a prespecified analytical framework informed by Directed Acyclic Graphs (DAGs). By integrating this causal logic with multivariable mixed-effects regression, we aim to identify the independent factors associated with publication lag, thereby informing potential strategies to optimize the editorial workflow and accelerate the publication of high-quality medical evidence.

## Methods

The present research protocol describes a meta-epidemiological study, which is based on systematic reviews with meta-analyses, aiming to identify research and publication characteristics associated with publication time lag. The protocol for this study has been registered on the Open Science Framework (OSF) (Registration DOI: 10.17605/OSF.IO/CJTKU; available at: https://osf.io/cjtku). which will be reported in accordance with the PRISMA 2020 statement and relevant methodological reporting guidelines.

### Literature search

We will search PubMed, EMBASE (using Ovid) and Web of Science to identify interventional systematic reviews with meta-analyses published in the Lancet, JAMA, NEJM, The BMJ, and the CDSR of meta-analyses from January 1, 2023 to December 31, 2025. We will restrict the search to the target journals using the journal source field, combining study design filters with relevant subject headings and keywords. The publication date limit will also be applied. The complete search strings for each database are provided in the Supplementary material (see Supplementary Tables S1–S3) (14).

### Eligibility criteria

We will include all interventional meta-analyses. Detailed inclusion and exclusion criteria are presented in Table 1.

**Table 1:** Selection criteria considering participants, intervention, comparison and outcomes.

Domain	Eligibility criteria
Participants	Systematic reviews with meta-analyses of clinical interventions
Intervention	Interventional studies evaluating therapeutic, preventive, or screening measures (e.g., pharmacotherapy, surgery, or psychotherapy).
Comparison	No restrictions on comparators (including placebo, no intervention, usual care, or active drugs)
Outcomes	Studies reporting results of quantitative synthesis (meta-analysis)
Study design	Systematic reviews with meta-analyses including only randomized controlled trials (RCTs)
Exclusion	Narrative reviews, editorials, commentaries, methodological papers, conference abstracts, and study protocols. Network meta-analyses (NMAs) and umbrella reviews not involving interventional comparisons, or studies where the interventional nature of the evidence synthesis could not be clearly determined. Studies where all key chronological dates are missing and remain irretrievable after supplementary retrieval efforts.

### Screening and selection

Two researchers (Jia Song, Qin Wang) will independently screen all resulting articles from the literature search to assess their eligibility, and disagreements will be resolved after consulting the third researcher (Deying Kang). The reasons for exclusion will be recorded and a summary of these reasons will be presented.

### Data extraction and consistency assessment

Two researchers (Jia Song, Qin Wang) will construct a data extraction form 5% of all included meta-analyses were randomly selected for double data extraction to ensure continual agreement between the reviewers. For this purpose, a random sequence without duplicate numbers was generated with the software R. Consistency will be assessed using Cohen’s Kappa coefficient, where a value of 0.80 (15). If the inter-rater agreement falls below the threshold, the sampling proportion will be increased. Any discrepancies will be resolved by consulting the third researcher (Deying Kang).

### Outcomes and definition of publication lag

#### Primary outcome

The primary outcome was publication time lag, which will be calculated in days as the difference between the date of last search and online publication date (5). If the online date is unavailable, the issue publication date will be used. The source and hierarchy of the date will be recorded in the data dictionary. In our primary analytical approach, publication lag will be treated as a continuous variable (measured in days). Subsequently, to facilitate methodological and clinical interpretation against established evidence-synthesis standards, the continuous lag will be transformed into categorical outcomes using the following two distinct classification systems.

### Classification criteria based on AMSTAR 2

Based on the AMSTAR 2 criterion, which requires the literature search to be performed within 24 months prior to publication to be considered comprehensive, studies will be classified into “Timely” (lag < 24 months) and “Untimely” (lag ≥ 24 months) categories (10).

### Classification criteria based on Cochrane

Based on MECIR Standard, which requires a search update within 12 months and recommends 6 months, we will categorize timeliness as: “High Currency” (lag < 6 months; adhering to best practices with minimal bias risk); “Acceptable Currency” (lag 6–12 months; meeting minimum mandatory standards); and “Low Currency/Outdated” (lag > 12 months; failing to meet standards with substantial risk of time-lag bias) (9).

#### Secondary outcomes

1. Submission-to-acceptance lag will be calculated in days as the difference between the date of submission and acceptance date.
2. Acceptance-to-online publication lag will be calculated in days as the difference between acceptance date and online publication date.
3. Submission-to-online publication lag will be calculated in days as the difference between the date of submission and online publication date.

These time lags will be treated as continuous variables and summarized as medians with interquartile ranges (IQRs).

### Study characteristics and variables

Regarding bibliographic attributes, we will extract the first author’s name, the corresponding author’s country, and the specific journal of publication. To quantify the publication lag, we will record the online publication date and the date the literature search ended.

The primary comparative factor is journal type, categorized as the CDSR or top-tier general medical journals. Other comparative factor includes methodological quality, open science practices, and interpretative judgment and discussion. To assess the methodological integrity of the evidence synthesis, we will examine adherence to the PRISMA statement, AMSTAR 2 guidelines, and the use of a standard PRISMA flowchart (16, 17). Quality assessment metrics will include the application of ROB 2 (18), GRADE (19), 95% prediction intervals, and the assessment of publication bias (20, 21). We will also record the meta-analytical models (fixed-effect vs. random-effects) used in the primary analysis. Finally, we will examine factors related to transparency and the final phase of dissemination. This includes open science practices, such as data sharing statements (22, 23). Within the interpretative judgment and discussion domain, we will evaluate how authors explained between-study heterogeneity (24, 25), whether they mentioned the concept of Minimal Clinically Important Difference (MCID), and their exploration of the clinical relevance underlying the statistical findings (26, 27).

We hypothesize that potential confounders include funding sources, research team characteristics, study types and research fields, and study complexity may serve as potential confounders in the relationship between the journal type and publication lag. To account for the influence of the research environment on publication efficiency, we will extract funding sources (e.g., non-profit vs. for-profit) (5). Additionally, research team characteristics will be assessed by recording the number of authors and their institutional affiliations, specifically identifying clinical and methodological/statistical departments. To characterize the research domain, target diseases will be classified according to the ICD-11chapter structure, and the focus of the meta-analysis (efficacy, safety, or both) will be recorded (28, 29). To measure study complexity, which acts as a proxy for the analytical workload, we will extract the number of databases searched, the number of RCTs included, the total pooled sample size, and the number of primary and secondary outcomes. We will also document technical details such as data types (continuous vs. binary), effect measures applied (e.g., RR, OR, MD), and specific intervention types (30).

### Statistical analysis

A sample size calculation is usually not performed in systematic reviews of reviews or meta-epidemiological studies. Based on preliminary searches, we assumed to include a total of 250 to 300 reviews which is comparable to or even exceeds the sample size of other methodological studies (31–37). Although a formal *a priori* power calculation is not applicable for this exploratory observational design, the anticipated sample size of 250 to 300 reviews safely satisfies the “Events Per Variable (EPV)” rule of thumb for robust multivariable modeling (38, 39).

**Figure 1:**
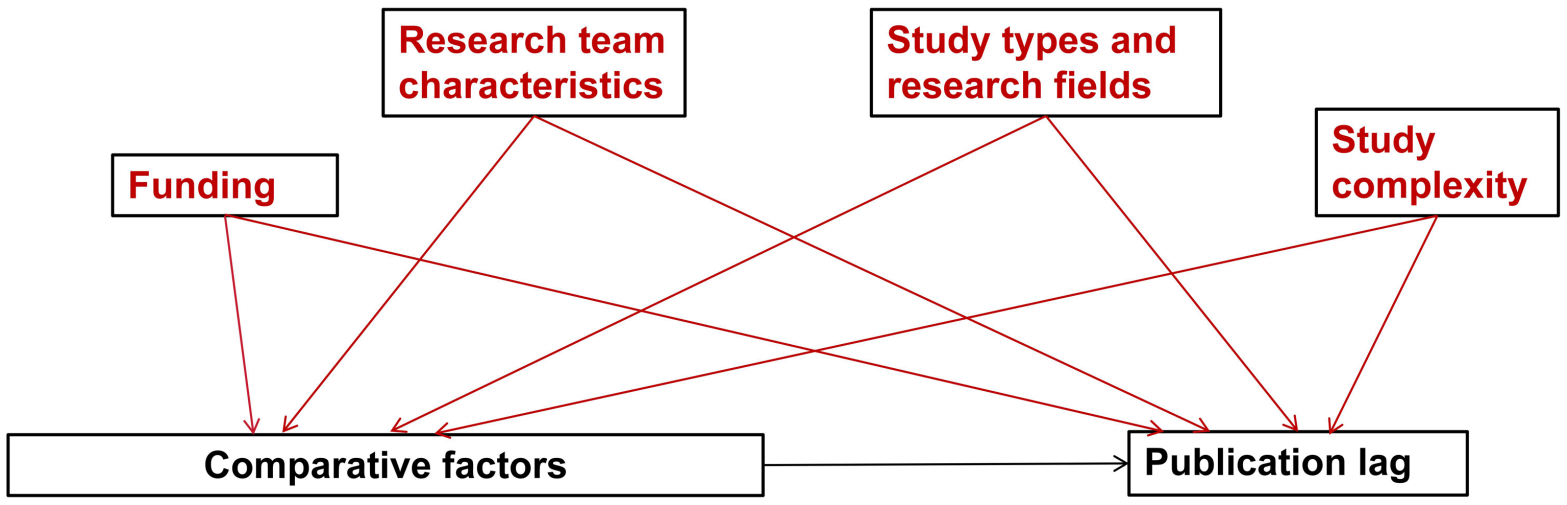
The DAG-informed conceptual framework.

To ensure methodological transparency, variable selection was guided by a prespecified DAG-informed conceptual framework (Figure 1). For our primary analysis, we model journal type as the key exposure and publication lag as the outcome, while adjusting for the aforementioned confounders. Subsequently, we will adopt the same DAG-based methodological principle across separate, parallel analyses. In these dedicated models, methodological integrity, open science practices, and interpretative judgment will be reframed as distinct exposures in relation to publication lag to disentangle their independent associations with evidence timeliness.

Categorical variables will be described using counts and percentages. Continuous variables will be presented as medians and IQRs due to the expected skewed distribution. The results will be presented stratified by journal, year of publication, disease area, and the presence of pre-registration or data sharing statements.

Regarding the primary outcome “publication lag,” modeling analyses will be performed based on the AMSTAR 2 criteria (binary: timely/not timely) and the Cochrane MECIR standards (ternary: high/acceptable/outdated). For the binary outcome, multivariable logistic regression models will be fitted to estimate ORs and 95% confidence intervals(95%CIs). For the ternary outcome, ordered logistic regression will be prioritized; however, if the proportional odds assumption is violated upon testing, multinomial logistic regression will be used instead. To account for within-journal correlation, our primary analytical strategy will employ mixed-effects models with journals specified as a random intercept. Adjustment via journal fixed effects will be conducted exclusively as a supplementary sensitivity analysis.

The variables will be derived from a pre-specified variable dictionary (see Table 2), covering publication characteristics (e.g., journal, year of publication, impact factor, and citation counts), research scope (e.g., disease area and efficacy/safety orientation), and methodological and open science characteristics (e.g., PRISMA/AMSTAR 2 statement, ROB2, GRADE, protocol pre-registration, and data sharing). The full model will be constructed by combining a prior knowledge with results from univariate analyses. Collinearity will be assessed using a correlation matrix; variables with a Spearman correlation coefficient ≥ 0.7 will not be included in the same model. Variable selection will be primarily based on a prior knowledge, supplemented by backward elimination and the minimization of the Akaike Information Criterion (AIC). The consistency of results across different selection strategies will be reported (40).

**Table 2:** The extracted variables.

Category	Extracted items
Basic characteristics	
	1. First author
	2. Corresponding author’s country of the primary affiliation
	3. Journal of publication
	4. Online publication time
	5. Search date
Funding	
	6. Funding: (a) self-funded, (b) non-profit organization funding, (c) for-profit organization funding, (d) mixed self-funded and non-profit, (e) mixed non-profit and for-profit, and (f) no funding information/not reported
Research team characteristics	
	7. Number of authors
	8. Institutional affiliations: (a) none, (b) at least one clinical department, (c) at least one methodological/statistical department (e.g., Center for Evidence-Based Medicine, Department of Epidemiology, or Biostatistics), (d) at least one clinical and one methodological department, (e)no specific department/unclear
Study types and research fields	
	9. Target diseases
	10. Focus of the meta-analyses: (a) efficacy assessment only, focusing on beneficial outcomes of the interventions; (b) safety assessment only, focusing on harmful outcomes or adverse events; and (c) concurrent efficacy and safety assessment, where both efficacy and safety indicators are listed as co-primary outcomes or discussed with equal weight in the conclusion.
Study complexity	
	11. Number and names of the databases
	12. Number of RCTs included
	13. Pooled sample size
	14. Number and specific names of the primary outcomes
	15. Number and specific names of the secondary outcomes
	16. Specific types of interventions in the experimental and control groups
	17. Baseline characteristics (e.g., age and sex)
	18. Data type (continuous or binary)
	19. Effect measures applied (e.g., RR, OR, HR, MD or SMD)
Methodological quality	
	20. PRISMA statement or its extensions
	21. AMSTAR 2 methodological guidelines
	22. ROB 2
	23. Proportion of studies classified as “high risk,” “low risk,” and “some concerns/unclear”
	24. GRADE
	25. Standard PRISMA flowchart
	26. Between-study heterogeneity
	27. Assess publication bias or small-study effects
	28.95% prediction intervals
	29. Sensitivity analyses
	30. Meta-analytical model applied for the primary analysis: (fixed-effect model, random-effects model, or both)
Open science	
	31. Protocol pre-registration available
	32. Data sharing statement
Interpretative judgment and discussion	
	33. Explained the between-study heterogeneity: (a) not discussed, (b) clinical heterogeneity only (e.g., patient characteristics or differences in interventions), (c) methodological heterogeneity only (e.g., risk of bias or study design), and (d) both clinical and methodological heterogeneity.
	34. Mentioned the concept of MCID
	35. Explored the clinical relevance underlying the statistical findings

To address potential measurement heterogeneity arising from the substitution of issue publication dates for unavailable online-first dates, we will perform a pre-specified sensitivity analysis. This analysis will be restricted to a subset of articles with confirmed electronic publication dates. By re-running our primary multivariable ordered logistic regression model on this subset, we aim to evaluate whether the inclusion of substituted dates significantly influences the estimated predictors of publication delay. To assess the temporal stability of our findings, sensitivity analyses will be conducted by stratifying the data across the distinct journal type and publication years (2023–2025).

For included studies with partial missingness (e.g., missing specific days or months but possessing at least two anchor dates), we will prioritize retrieving information from the main text, Supplementary materials, appendices, or methods sections. If the information remains unavailable, the data will be recorded as missing. Assuming the missing data mechanism is Missing at Random (MAR), we will handle missing values using multiple imputation. To maximize the accuracy of the imputed values, the imputation model will incorporate the primary outcome variables as well as key auxiliary predictors, including the journal name, year of publication, and protocol pre-registration status. All tests will be two-sided. We will set the level of statistical significance at *p* value < 0.05. All multivariable analyses will be performed using R version 4.3.2.

## Discussion

This study aims to assess the publication time lag in systematic reviews with interventional meta-analyses, and to identify potential factors influencing these delays. While systematic reviews with meta-analyses serve as the cornerstone of clinical practice guidelines, the interval between the “date of last search” and “online publication” may render the evidence partially outdated upon release. This latency compromises the applicability of the findings for evidence users and complicates decision-making regarding the necessity for updates. Despite the critical implications of delayed evidence updates, the extent and associated factors of this lag in top-tier general medical journals have yet to be rigorously quantified under a unified methodological framework. To date, existing literature has predominantly relied on descriptive analyses, failing to provide a standardized assessment of the problem’s scale and determinants. Therefore, the primary aim of this study is to conduct a comprehensive systematic assessment of the publication timeliness of interventional meta-analyses published between 2023 and 2025, and to explore the potential factors influencing these delays.

To address these gaps, our study focuses on standardizing definitions and ensuring a clear analytical framework. First, by combining timeliness standards from Cochrane MECIR and AMSTAR 2, we will create a structured grading system. This provides a meaningful “timeliness judgment” that complements the actual time data. Second, we will compare CDSR with top-tier general medical journals to explore differences in timeliness across different publishing systems, offering insights into why delays happen and how to improve. Finally, we will build a multivariable model including publication characteristics, methods, and team structure. This allows us to identify the associated factors of publication lag while controlling for other factors, providing solid evidence for future improvement.

This meta-epidemiological study aims to provide reproducible, quantitative evidence regarding publication lags in systematic reviews with meta-analyses within top-tier general medical journals and the CDSR, presenting their association patterns with multidimensional characteristics under a unified framework. Our findings will inform the management of evidence synthesis workflows and enhance reporting transparency within the evidence ecosystem (41). Specifically, the study offers an actionable framework for journals to standardize process disclosure, and for guideline developers and clinical end-users to assess evidence currency and applicability. Ultimately, this work seeks to facilitate a paradigm shift from empirical assumptions about the “risk of obsolescence” to data-driven empirical assessments, laying the groundwork for future validation and improvement interventions across broader journals, disciplines, and specific procedural stages.

### Strengths and limitations of this study

This study evaluates publication timeliness within two high-impact evidence-synthesis ecosystems: top-tier general medical journals and the CDSR.

Departing from traditional descriptive approaches, this study utilizes a Directed Acyclic Graph (DAG)-based causal framework to systematically identify and select potential confounders and mediators. We systematically accounted for a vast array of potential confounders and mediators. This broad variable assessment ensures that our findings provide robust, actionable insights for authors and editors to optimize the transition from evidence synthesis to public dissemination.

The focus is purposively restricted to top-tier general medical journals and the CDSR to evaluate high-impact evidence ecosystems, but findings may not be generalizable to specialty journals where publication dynamics and resource constraints differ.

The analysis relies on publicly available dates and cannot distinguish between delays originating from author-led revisions versus those inherent to the editorial peer-review process.
