# Supplementary material for: Publication lag in interventional, RCT-based meta-analyses within top-tier general medical journals and the CDSR: a protocol for a meta-epidemiological study
Source: Front Med (Lausanne). 2026 Apr 24;13:1803578. doi: 10.3389/fmed.2026.1803578 (PMC13153117; doi:10.3389/fmed.2026.1803578)
Supplement: Supplementary file 1 [file Data_Sheet_1.pdf]

Supplementary Material

Supplementary Table 1 EMBASE(Ovid) Search keywords

#	Search keywords
1	Meta-Analysis as Topic/
2	meta-analysis.pt.
3	(meta-analy$ or metanaly$).tw.
4	(systematic adj (review$1 or overview$1)).tw.
5	1 or 2 or 3 or 4
6	("New England Journal of Medicine" or "N Engl J Med").jn.
7	("Lancet" or "The Lancet").jn.
8	("JAMA" or "Journal of the American Medical Association").jn.
9	("BMJ" or "British Medical Journal").jn.
10	("Cochrane Database of Systematic Reviews").jn.
11	6 or 7 or 8 or 9 or 10
12	5 AND 11
13	limit 12 to yr="2023 - 2025"
14	limit 13 to (english language and humans)

Supplementary Table 2 PubMed Search keywords

#	Search keywords
1	"meta-analysis"[Publication Type]
2	"meta-analysis as topic"[MeSH Terms]
3	"systematic reviews as topic"[MeSH Terms]
4	"meta-analysis"[Title/Abstract]
5	"meta-analy*"[Title/Abstract]
6	"systematic review"[Title/Abstract]
7	"systematic overview"[Title/Abstract]
8	1 OR 2 OR 3 OR 4 OR 5 OR 6 OR 7
9	"N Engl J Med"[Journal]
10	"Lancet"[Journal]
11	"JAMA"[Journal]
12	"BMJ"[Journal]
13	"Cochrane Database Syst Rev"[Journal]
14	9 OR 10 OR 11 OR 12 OR 13
15	8 AND 14
16	15 AND 2023/01/01:2025/12/31[Date - Publication]

Supplementary Table 3 Web of science Search keywords

#	Search Queries (Advanced Search Syntax)
1	TS=("meta-analysis")
2	TS=("meta-analy*")
3	TS=("systematic review")
4	TS=("systematic overview")
5	#1 OR #2 OR #3 OR #4
6	SO=("New England Journal of Medicine")
7	SO=("Lancet")
8	SO=("JAMA" OR "Journal of the American Medical Association" OR "JAMA-Journal of the American Medical Association")
9	SO=("BMJ" OR "British Medical Journal" OR "BMJ-British Medical Journal")
10	SO=("Cochrane Database of Systematic Reviews")
11	#6 OR #7 OR #8 OR #9 OR #10
12	#5 AND #11
13	#12 AND DOP= (2023-01-01 TO 2025-12-31)
